# Supplementary material for: Synchronous quantitative analysis of chiral mesostructured inorganic crystals by 3D electron diffraction tomography
Source: Nat Commun. 2022 Sep 29;13:5718. doi: 10.1038/s41467-022-33443-1 (PMC9522932; doi:10.1038/s41467-022-33443-1)
Supplement: Supplementary file 1 — Supplementary Information [file 41467_2022_33443_MOESM1_ESM.pdf]

Supplementary Information

Synchronous Quantitative Analysis of Chiral Mesostructured Inorganic Crystals by 3D Electron Diffraction Tomography

Jing Ai[1], Xueliang Zhang[2], Te Bai[2], Qing Shen[2], Peter Oleynikov[3], Yingying Duan[1], Osamu Terasaki[3], Shunai Che[1,2] and Lu Han[1]*

[1]School of Chemical Science and Engineering, Tongji University, 1239 Siping Road, Shanghai, 200092, China.

[2]School of Chemistry and Chemical Engineering, Frontiers Science Center for Transformative Molecules, State Key Laboratory of Composite Materials, Shanghai Key Laboratory for Molecular Engineering of Chiral Drugs, Shanghai Jiao Tong University, 800 Dongchuan Road, Shanghai, 200240, China.

[3]School of Physical Science and Technology, Centre for High-resolution Electron Microscopy and Shanghai Key Laboratory of High-resolution Electron Microscopy, ShanghaiTech University, Shanghai 201210, China.

*e-mail: luhan@tongji.edu.cn.

Inventory of supporting information:

1. **Supplementary Figures 1-32**
2. **Supplementary Tables 1-8**

1. Supplementary Figures

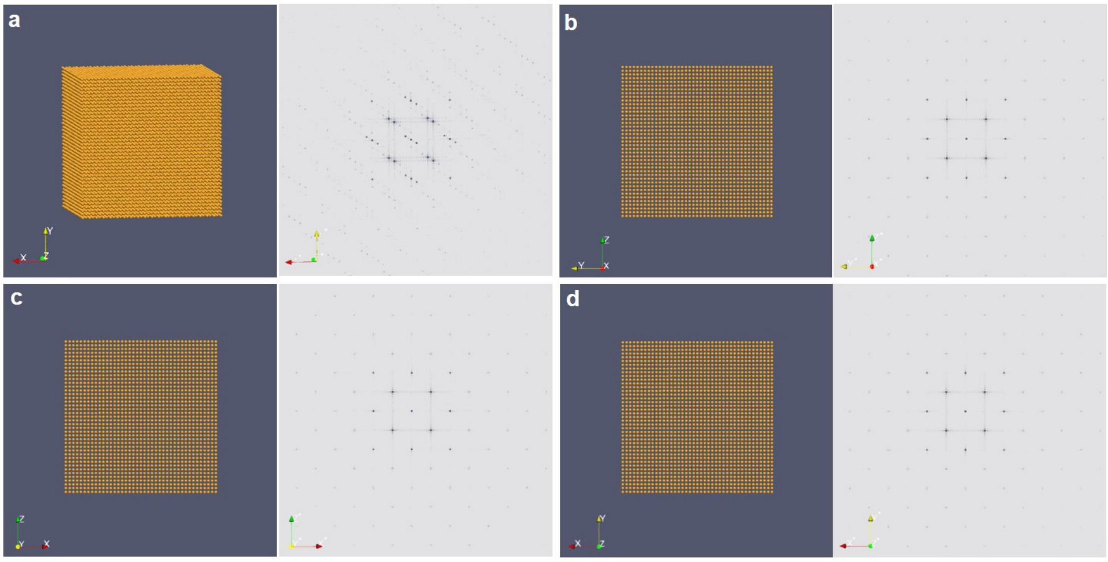

Supplementary Figure 1. Structural model of Au single crystal and the corresponding reciprocal space. a-d, The Au single crystal model observed from different orientations and the corresponding Fourier transform shown on the right. The repeating units are set to be 30, 30, 30 for the *x*-, *y*-, *z*-axes, respectively. Due to the limitation of the crystal size, the diffused intensity distribution of the diffraction spots can be observed. The corresponding three-dimensional (3D) electron diffraction (ED) data is shown in the **Supplementary Movie 1**.

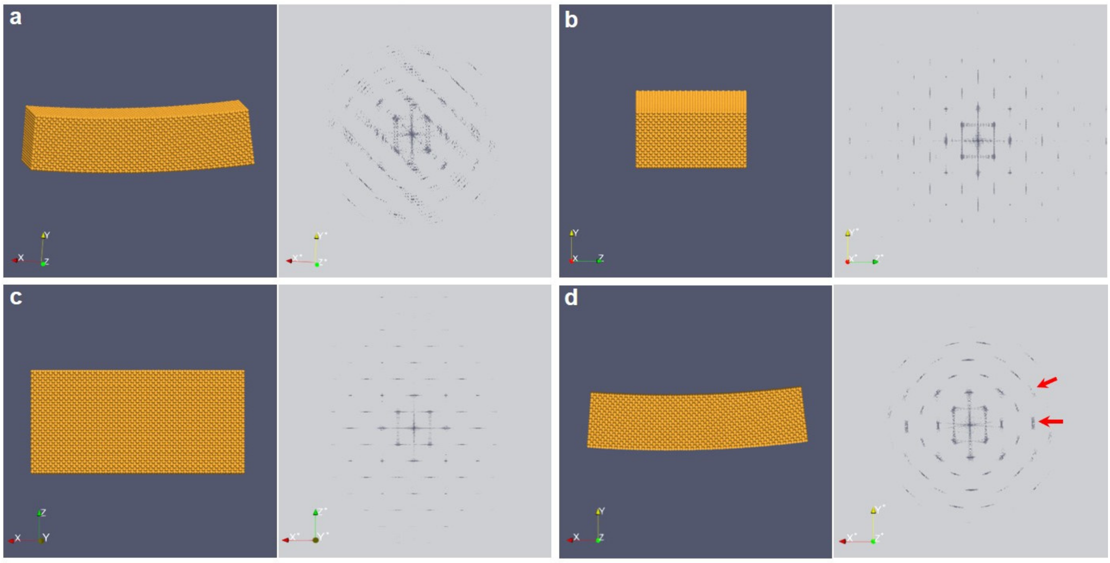

Supplementary Figure 2. Structural model of bent Au crystal and the corresponding reciprocal space. a-d, The bent Au crystal structure model and its Fourier transform observed from different orientations. The repeating units are set to be 40, 10, 20 for the *x*-, *y*-, *z*-axes, respectively. The bending is along *z*-axis with the bending angle of 10°. The red arrows indicate the modulation of stretched arched-shape diffraction spots corresponding to the bending angle and orientation. The corresponding 3D ED data is shown in the **Supplementary Movie 2**.

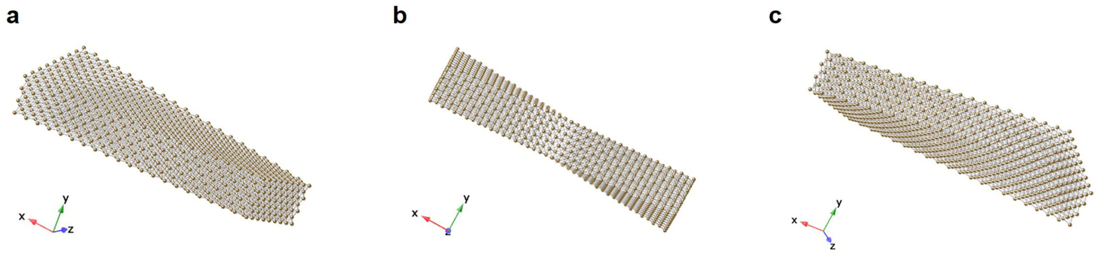

Supplementary Figure 3. Schematic drawing of chiral structure with twisted Au lattices from different orientations. a-c, The twisted Au lattices observed from three different orientations, respectively. The repeating units are set to be 20, 3, 8 for the *x*-, *y*-, *z*-axes, respectively. The twisted Au crystal was built by rotating the Au atoms by 0.245°/ Å along the *x*-axis and crystal lattice torsion along the *y*-axis and *z*-axis can be also observed simultaneously.

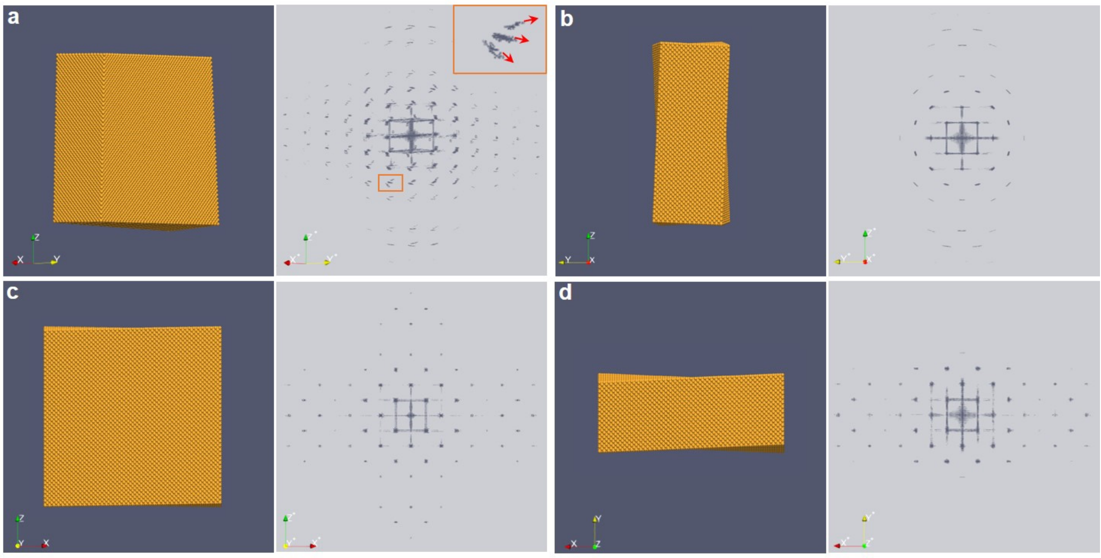

Supplementary Figure 4. Structural model of twisted Au crystal and the corresponding reciprocal space. a-d, Twisted Au crystal structure and its Fourier transform observed from different axes. The repeating units are set to be 40, 15, 40 for the *x*-, *y*-, *z*-axes, respectively. The twisted Au crystal was built by rotating the Au atoms by 0.0147°/ Å along the *x*-axis. The modulated diffraction spots not only form the arch-shaped intensity distribution (**b**) but also arranged in a rotational manner (**a**). The inset of **a** indicates a rotational arrangement of the typical arched-shape diffraction spots. The corresponding 3D ED data is shown in the **Supplementary Movie 3**.

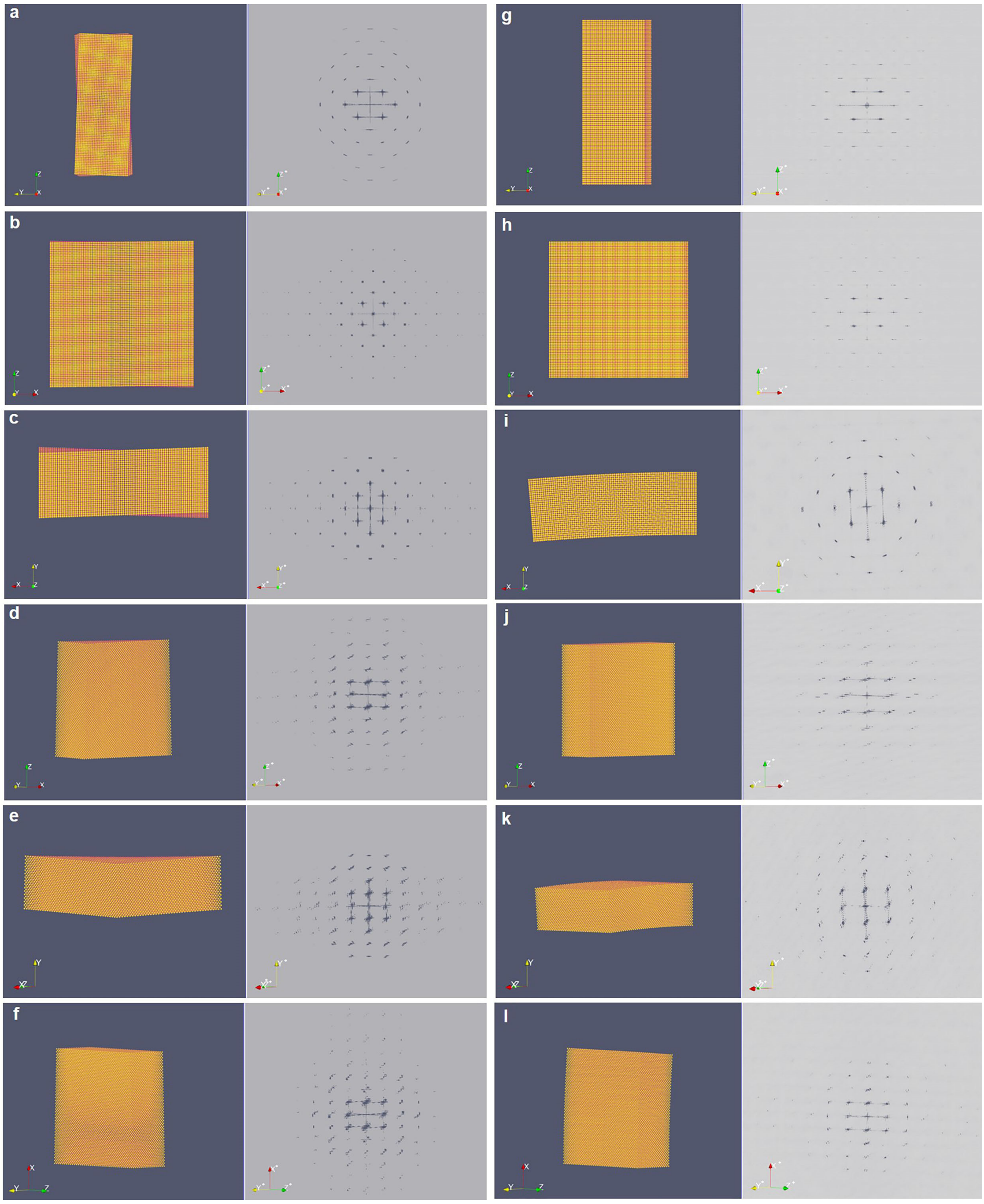

Supplementary Figure 5. Comparison of the modulated reciprocal space of twisting and bending crystal structures. a-f, Structural model of twisted Au crystal and the corresponding reciprocal space. The twisted Au crystal was built by rotating the Au atoms by 0.0147°/ Å along the *x*-axis. **g-l,** Structural model of bent Au crystal and the corresponding reciprocal space. The bending is along the *z*-axis with the bending angle of 5°. The repeating units for both structures were set to be 40, 15, 40 for the *x*-, *y*-, *z*-axes, respectively. It is worthy to note that, modulation in the bent crystal only exhibits the stretch diffraction intensity into arched-shape, while the diffraction spots in the twisted crystal show a rotational relationship.

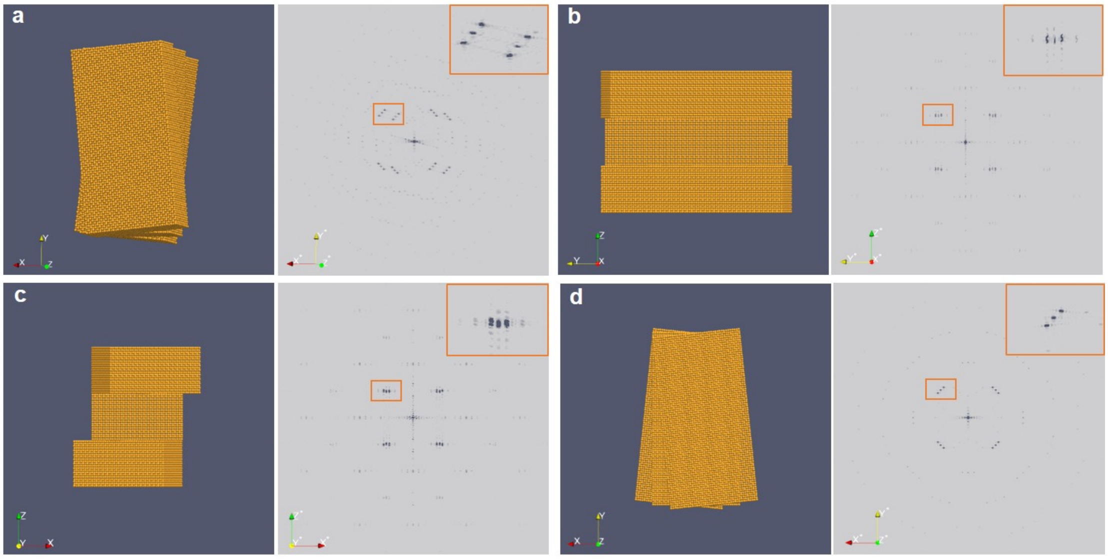

Supplementary Figure 6. Structural model of helically stacked Au nanocrystals and the corresponding reciprocal space. a-d, The helical stacking of Au nanocrystals was constructed by three layers of crystal with a rotation angel of 6°. The repeating units for each nanocrystal are 20, 40, 10 for the *x*-, *y*-, *z*-axes, respectively. Insets indicate the typical modulated diffraction spots corresponding to the stacked crystals. The corresponding 3D ED data is shown in the **Supplementary Movie 4**.

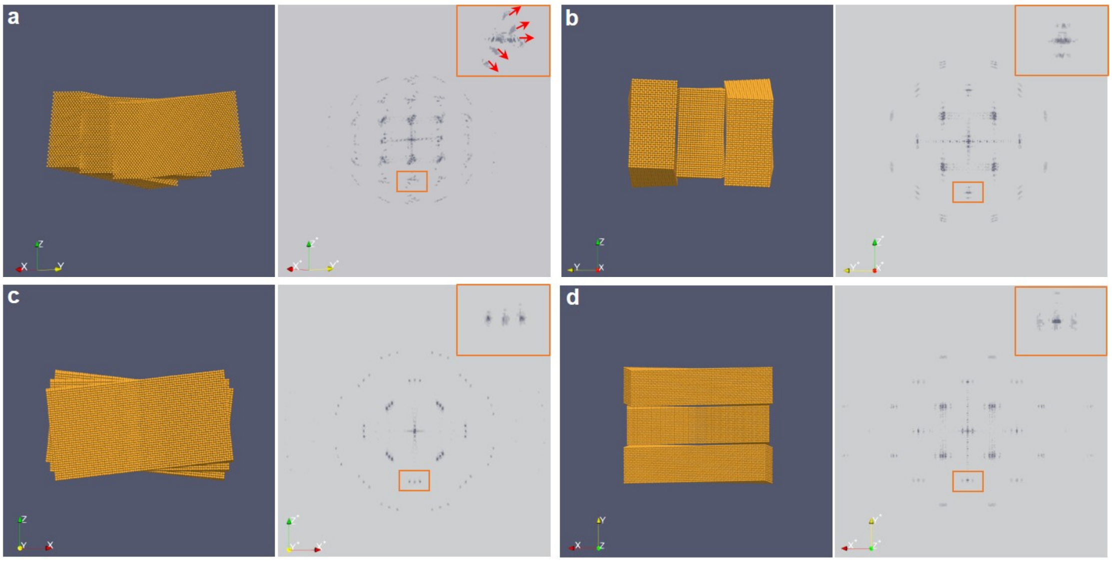

Supplementary Figure 7. Structural model of the chiral hierarchical mesostructure formed by the stacking of primary twisted nanocrystals and the corresponding reciprocal space. a-d, The assembly was constructed by three layers of twisted nanocrystals with 40, 10, 20 in size for the *x*-, *y*-, *z*-axes, respectively. The twisted Au nanocrystal was built by rotating the Au atoms by 0.0147°/ Å along the *x*-axis and the rotational angle between two adjacent nanocrystals is 6°. Insets indicate the typical modulation of three sets of diffraction spots corresponding to the three twisted nanocrystals. The red arrows (inset of **a**) suggest a rotational arrangement of the modulated diffraction spots. The corresponding 3D ED is shown in the **Supplementary Movie 5**.

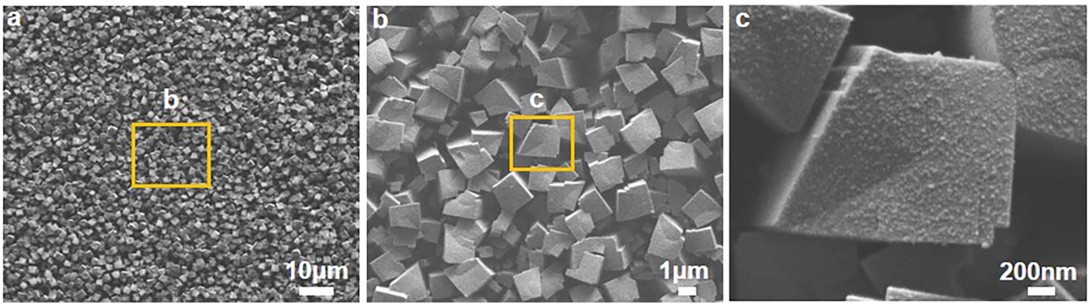

Supplementary Figure 8. SEM images of L-CNM taken at different magnifications. **a-c,** Step by step magnified SEM images of L-CNM. The enlarged areas are marked with yellow boxes, respectively.

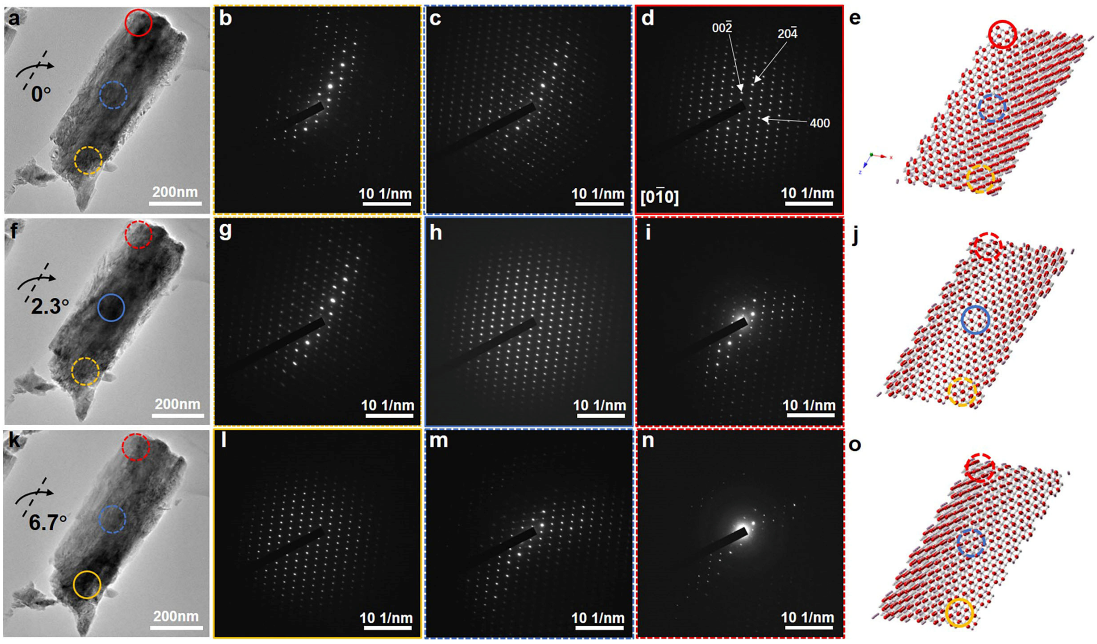

Supplementary Figure 9. Determination of primary chiral structure of D-CNM by manual tilting. a-o, TEM images and corresponding selected area ED (SAED) patterns of the rod-like particle as well as its schematic structural model of the right-handed form. When the top region of the particle is aligned to the $[0\bar{1}0]$ zone axis (**a-e**), the crystal becomes misaligned in the middle and bottom regions. By tilting the crystal along the $20\bar{4}$ reflection by 2.3° and 6.7°, respectively, the middle (**f-j**) and bottom (**k-o**) regions can be well aligned to the electron beam, indicating the rod-like particle is continuous twisted with a pitch length of 37.6 μm.

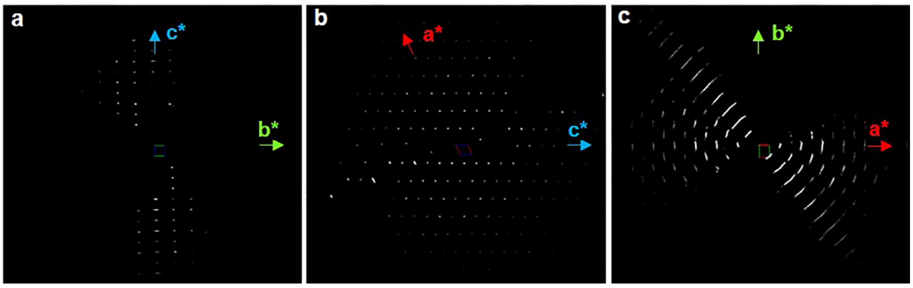

Supplementary Figure 10. The characteristic direction slices from the 3D ED patterns. a-c, The 0*kl*, *h*0*l*, *hk*0 slices taken from the reconstructed reciprocal lattice of the top area (A_1) part as shown in Fig. 2g in the main text of D-CNM, respectively.

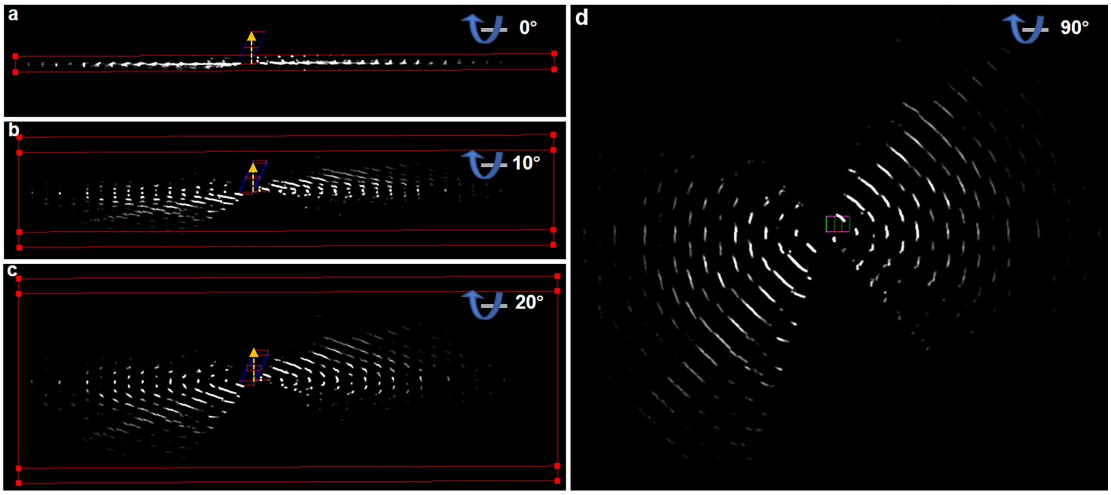

Supplementary Figure 11. Determination of the rotational axis of the top area (A_1) in the rod-like particle in D-CNM. a-d, The modulated arched-shape diffraction spots of the $h\bar{h}l$ slice. The direction perpendicular to the torsion axis shows the most obvious rotational intensity distribution. The yellow dotted line implies the rotational axis.

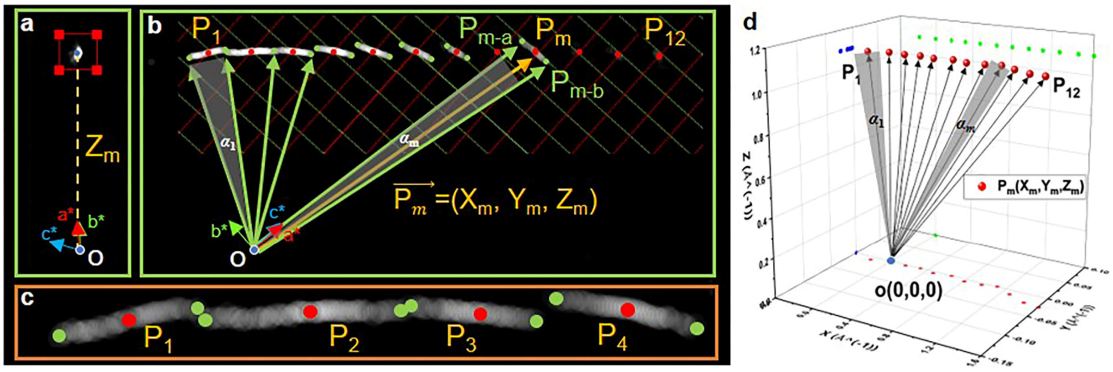

Supplementary Figure 12. Determination of the rotational angle by the intensity distribution of visibly diffraction spots. The $h(14-h)0$ diffractions (the enlargement permutation of green rectangle in Fig. 2m in the main text) cut from the reconstructed reciprocal lattice taken from A_1 part (Fig. 2g in the main text) of the rod-like D-CNM are shown in front view (**a**) and side view (**b**) and the magnified image characteristic region (**c**). P_m (X_m, Y_m, Z_m) represents the Cartesian coordinates of the diffraction spots (centers of the diffractions are marked by red dots and the two endpoints are marked by the green dots), and the green arrows represent the normal direction of the corresponding lattice planes. **d**, The 3D model diagram of the diffraction spots (P_m). α_m represents the rotational angle between two endpoints of P_m and the origin O. The results of P_m (X_m, Y_m, Z_m) coordinates are listed in Supplementary Table 2, The coordinates of endpoint defined as $P_{m\text{-}a}$ ($X_{m\text{-}a}$, $Y_{m\text{-}a}$, $Z_{m\text{-}a}$) and $P_{m\text{-}b}$ ($X_{m\text{-}b}$, $Y_{m\text{-}b}$, $Z_{m\text{-}b}$) are shown in Supplementary Table 3.

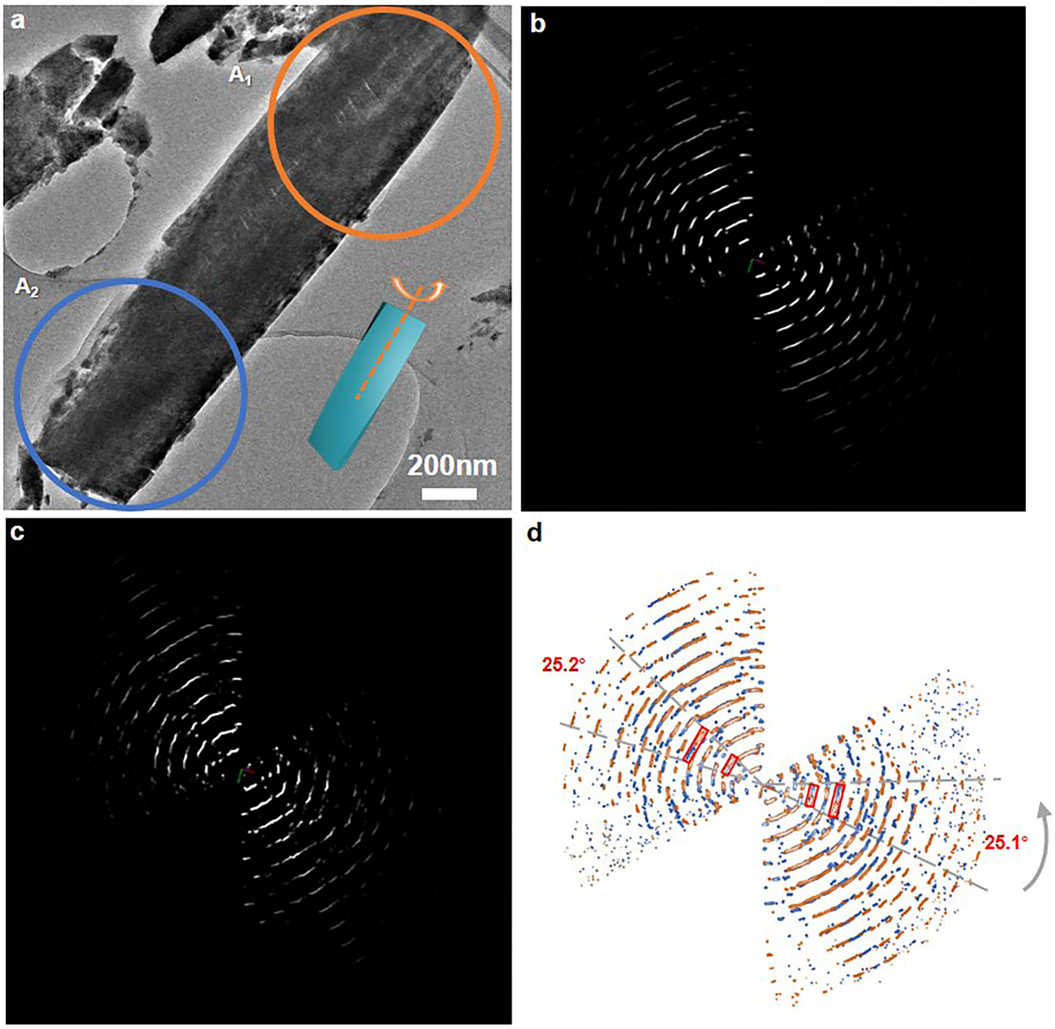

Supplementary Figure 13. Handedness determination of the rod-like particle by the 3D ED taken from two regions in D-CNM. a, TEM image of the rod-like particle of D-CNM and the orange and blue circles represent the two positions (A_1 and A_2) of SAED aperture for taking the 3D ED. The inset shows the schematic drawing of the twisted crystal. **b-c,** The modulated arched-shape diffraction spots of the $h\bar{h}l$ slice with rotational intensity distribution perpendicular to the rotational axis at the same orientation cut from the reconstructed reciprocal lattice of A_1 (**b**) and A_2 (**c**) in the rod-like particle. **d,** The superposed $h\bar{h}l$ slices from the two datasets taken from A_1 (orange) and A_2 (blue), respectively. The rotational intensity distribution of the two sets of 3D ED illustrates the lattice twisting from A_1 to A_2. The orientation of arched-shape diffraction spots from A_1 to A_2 shows a counterclockwise arranged manner, indicating a right-handed relationship of the rod-like particle in D-CNM. The complete intensity distribution (marked by the red rectangles) corresponding to the rod length from the farthest from A_1 to A_2. To make the results more accurate, the diffraction spots were selected in the middle part of the data collection range. The diffraction spots with relative complete connected intensity distribution into arch-shape were selected, forming the concentric circle amplification from inside to outside.

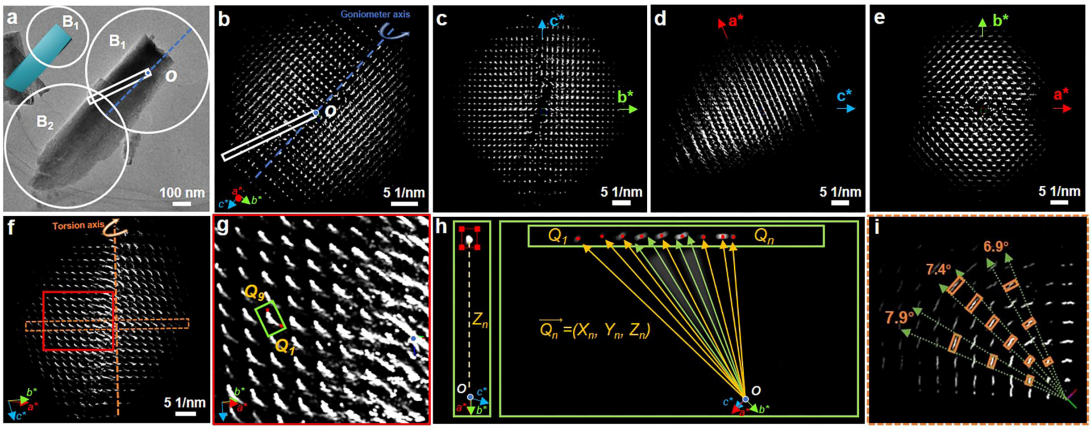

Supplementary Figure 14. Determination of primary chirality with twisted crystal lattices in L-CNM by 3D EDT. a-b, TEM image and 3D ED of the rod-like particle in L-CNM. The bule dotted line represents the goniometer axis and the white circles represent the two positions of the SAED aperture for taking the 3D ED. The top and bottom areas of the rod-like particle are denoted as B_1 and B_2, respectively. The inset of **a** shows a schematic drawing of the twisted crystal. **c-e,** 3D ED patterns of the particle taken from the B_1 part observed from the ***a****-, ***b****- and ***c****-axes, respectively. The amplitude of the diffraction is set to be square root for visualization. **f-g,** The enlargement permutation of the typical arched-shape ED distribution cut from the reconstructed reciprocal lattice of B_1 in L-CNM. The orange dotted line shown in **f** represents the position of the chiral rotational axis. **h,** The amplification of reflection (the green box in **g**). Q_n (X_n, Y_n, Z_n) represents the coordinates of diffraction spots (centers of the diffraction spots are marked by red dots) and the yellow arrows represent the normal direction of the corresponding lattice planes. **i,** Quarter amplification of arched-shape diffraction spots of the *hhl* slice (the orange rectangle of **f**) with rotational intensity distribution perpendicular to the rotational axis of the top area (B_1) in the rod-like particle.

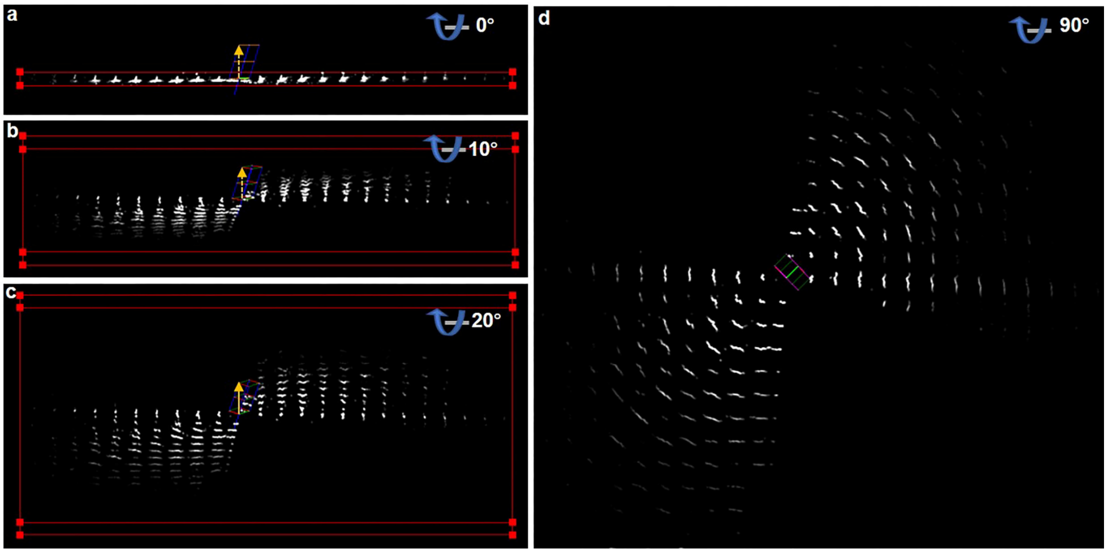

Supplementary Figure 15. Determination of the rotational axis of the top area (B_1) in the rod-like particle in L-CNM. a-d, The modulated arched-shape diffraction spots of the hhl slice. The direction perpendicular to the torsion axis shows the most obvious rotational intensity distribution. The yellow dotted line implies the rotational axis.

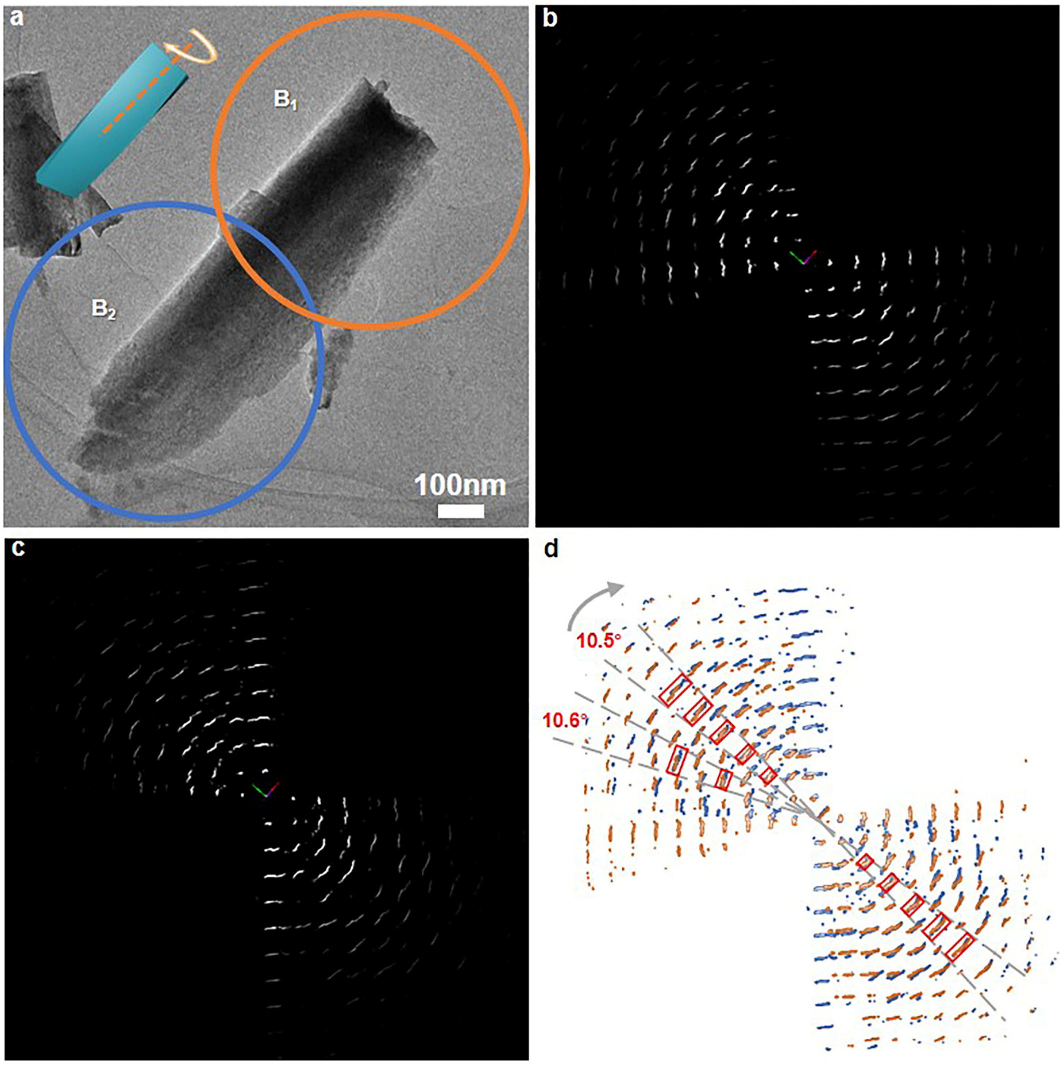

Supplementary Figure 16. Determination of handedness of the rod-like particle by the 3D ED patterns taken from the two regions in L-CNM. a, TEM image of the rod-like particle of L-CNM and the orange and blue circles represent the two positions (B_1 and B_2) of SAED aperture for taking the 3D ED. The inset shows the schematic drawing of the twisted crystal. **b-c,** The modulated arched-shape diffraction spots of the hhl slice with rotational intensity distribution perpendicular to the rotational axis at the same orientation cut from the reconstructed reciprocal lattice of B_1 (**b**) and B_2 (**c**) in the rod-like particle. **d,** The superposed hhl slices cut from the reconstructed reciprocal lattice of the two datasets taken from B_1 (orange) and B_2 (blue), respectively. The rotational intensity distribution of the two sets of 3D ED patterns illustrates lattice twisting from B_1 to B_2. The orientation of arched-shape diffraction spots from B_1 to B_2 shows a clockwise arranged manner, indicating a left-handed relationship of the rod-like particle in L-CNM. The crystal lattice torsion angle of the rod-like particle taken from L-CNM can be determined to be 10.55° along the normal direction of the $(20\bar{4})$ plane and the pitch length is calculated to be approximately 36.8 μm via the corresponding whole rod length of L-CNM measured to be ~1.1 μm from the farthest points from B_1 to B_2.

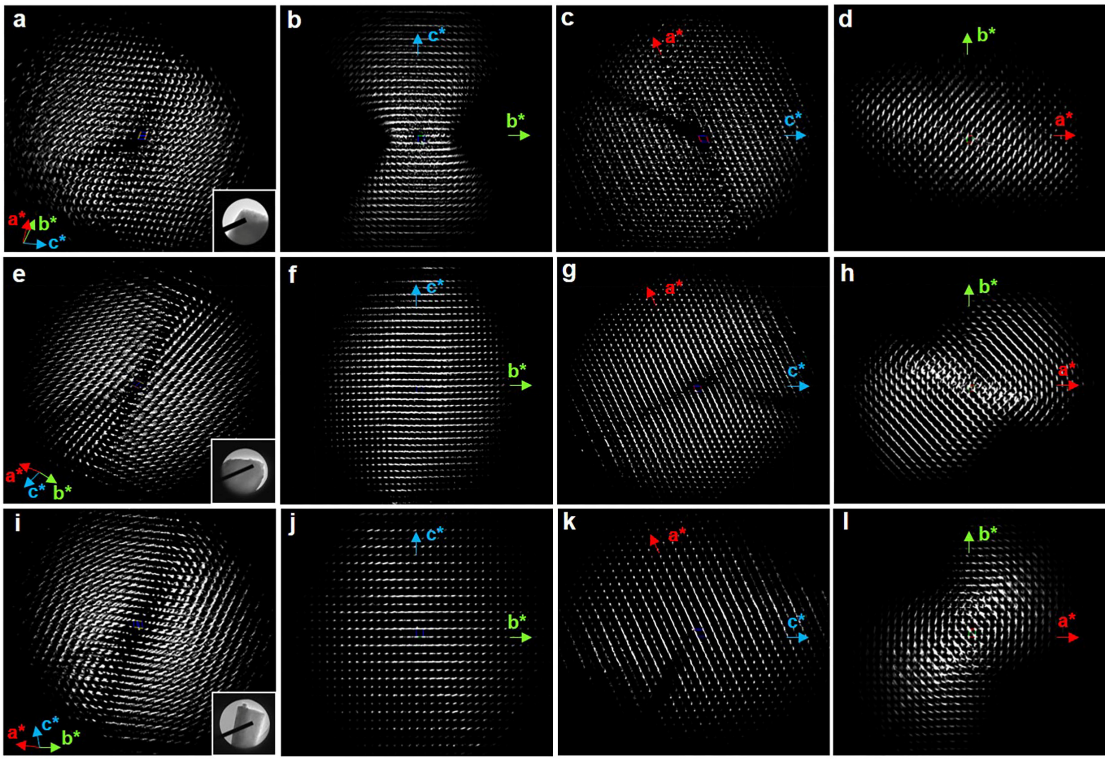

Supplementary Figure 17. 3D ED datasets of other samples in L-CNM and D-CNM, respectively. a-l, The whole 3D ED observed from different axes of L-CNM (**a-d**, **e-h**) and D-CNM (**i-l**), respectively. The tilting ranges are -50.3° to +44.2° for L-CNM-1 (**a-d**), -51.7° to +55.9° for L-CNM-2 (**e-h**), -50.2° to +50.0° for D-CNM-1 (**i-l**), respectively. The diffraction spots grayscale intensities are square roots of the corresponding measured values for the better visual experience during the publication.

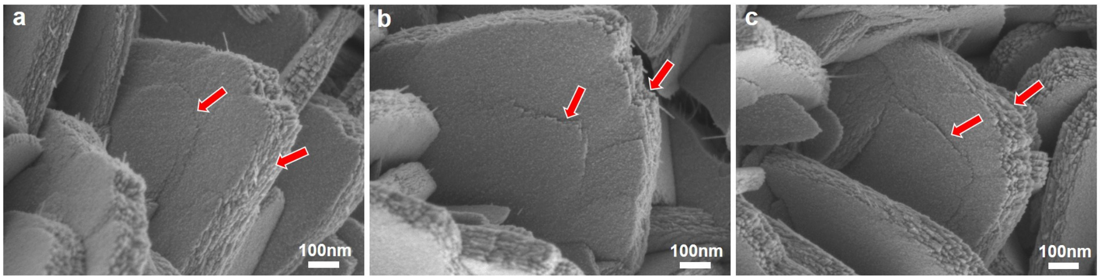

Supplementary Figure 18. SEM images of L-CTD with layered sheet-like structure. a-c, SEM images of the typical plate-like morphology samples with the layered sheet-like structure. The red arrows indicate the layered sheet-like steps in the plate-like particle.

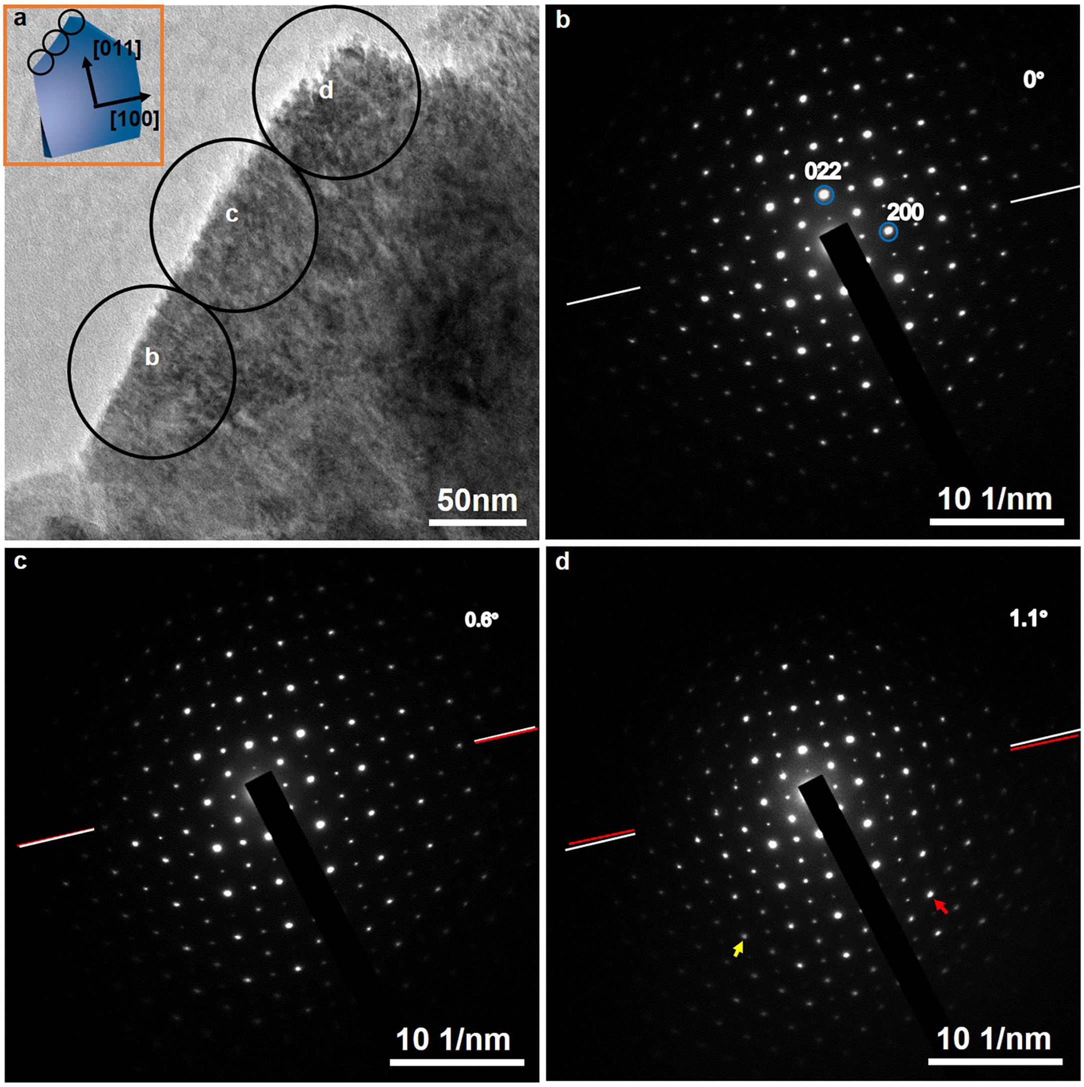

Supplementary Figure 19. TEM image and its corresponding SAED patterns taken from $[01\bar{1}]$ zone axis of the plate-like particle in L-CTD. a, TEM image of the plate-like particle in L-CTD and the black circles represent the three contiguous positions of SA aperture for taking the SAED patterns. The inset shows the schematic drawing of the plate-like particle. **b-d,** The corresponding SAED patterns. By the SAED patterns taken from three contiguous positions of the plate-like particle with the side length of ~270 nm, a rotational relationship with a tiny deflection of 1.1° was observed along the $[01\bar{1}]$ zone axis in a clockwise manner. Furthermore, the diffused intensity and split diffraction spots can be observed for the high index reflections (the red and yellow arrows of **d**), indicating a possible distortion of crystal lattices into the crystal bending or twisting arrangement.

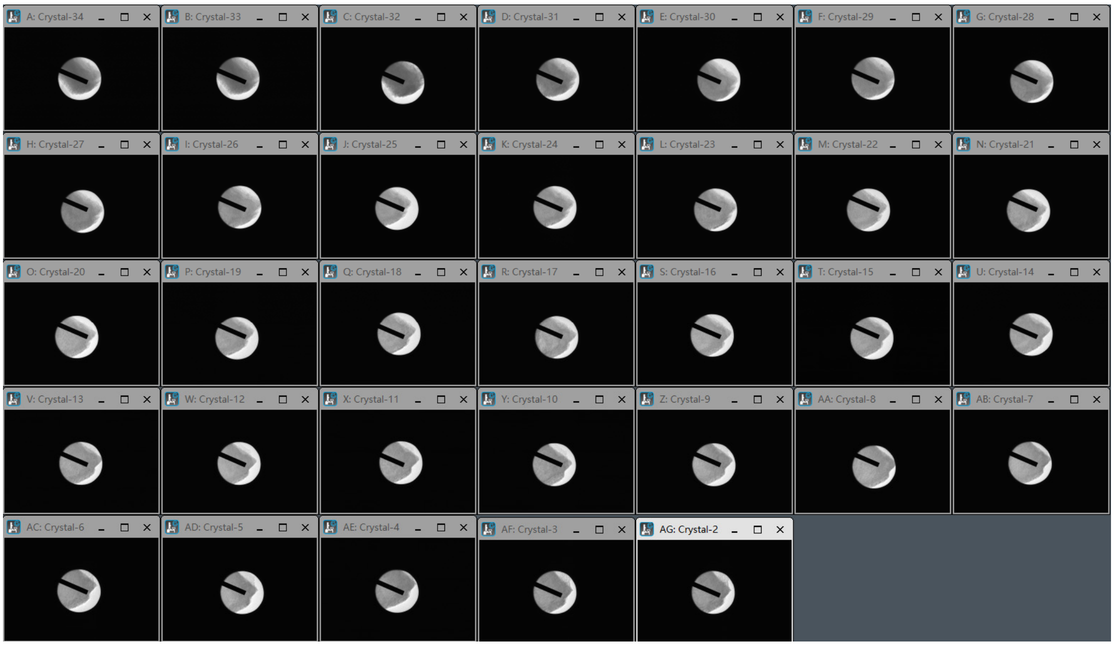

Supplementary Figure 20. The images automatically taken by the EDT-COLLECT software of the same L-CTD particle during the data collection process. Each image is taken for every goniometer tilting axis of 3°. During an hour of automatic acquisition, no damage or deformation of the crystal can be observed. The particle remains intact after 3D ED data collection and the ED pattern can be reproduced by going back to the original angle, indicating the crystal has good thermal stability.

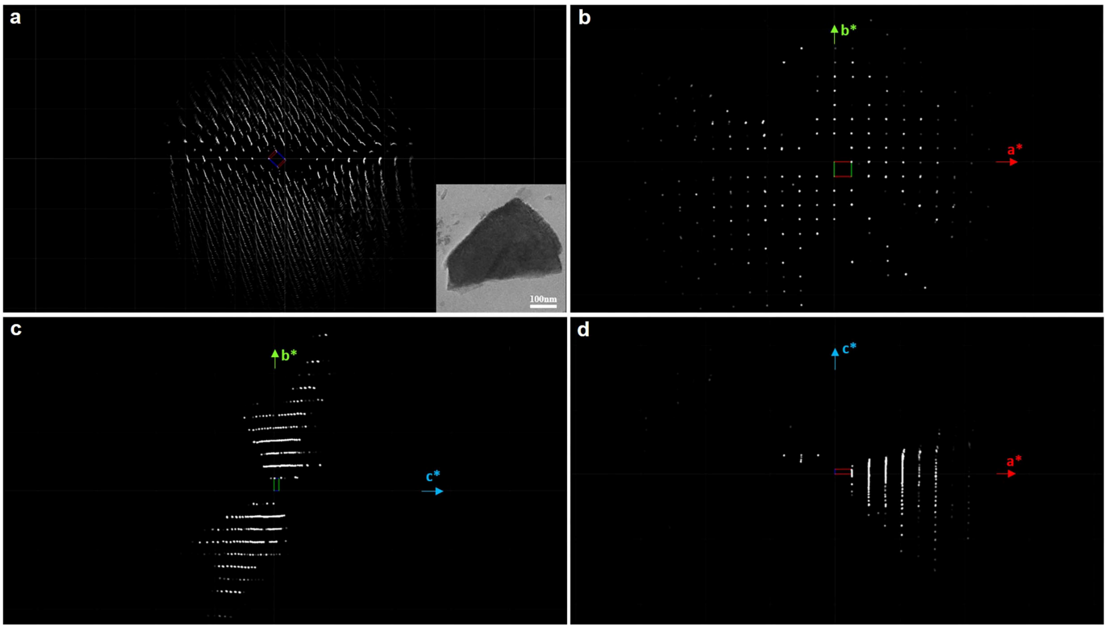

Supplementary Figure 21. The whole 3D ED and *hk*0, 0*kl*, *h*0*l* slices taken from the reconstructed reciprocal lattice of L-CTD. a, 3D ED data of the plate-like particle in L-CTD. Inset indicates the TEM image of the plate-like particle. **b-d,** The *hk*0, 0*kl*, *h*0*l* slices taken from the reconstructed reciprocal lattice of the plate-like particle.

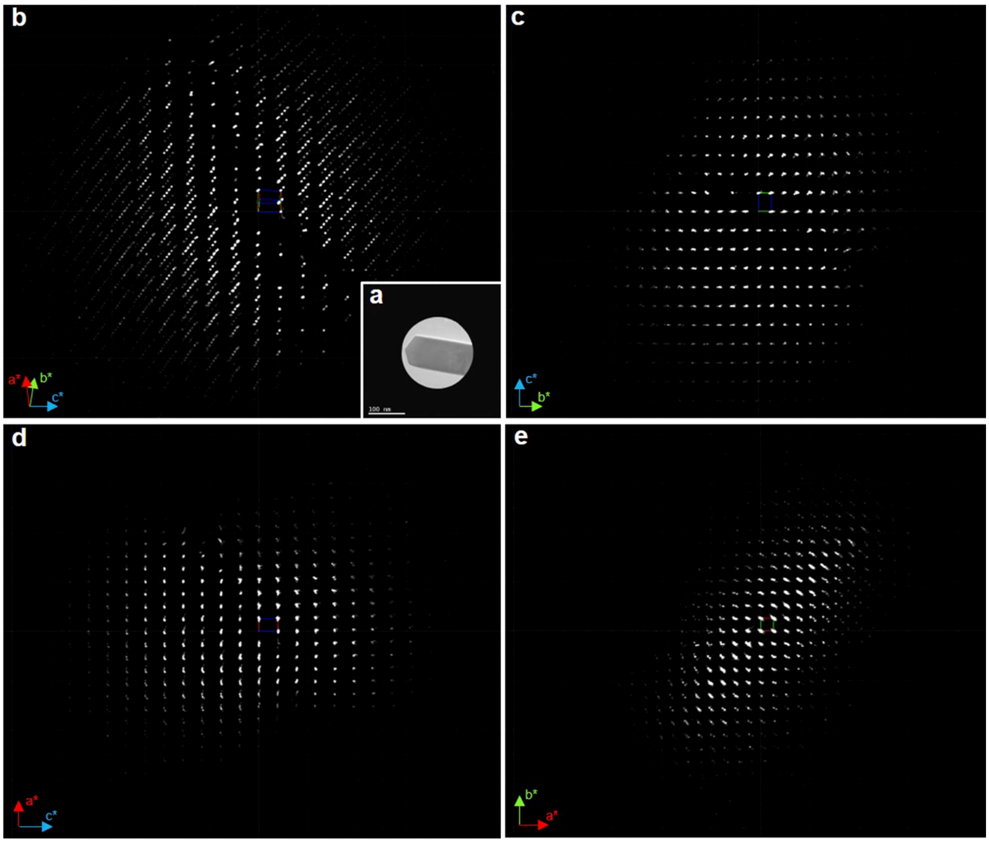

Supplementary Figure 22. 3D ED of commercial tin dioxide nanoparticles (CAS:18282-10-5). a, TEM image of the purchased tin dioxide particle. **b,** The whole 3D ED of the tin dioxide particle. **c-e,** 3D ED patterns of the purchased particle observed from the ***a****-, ***b****- and ***c****-axes, respectively.

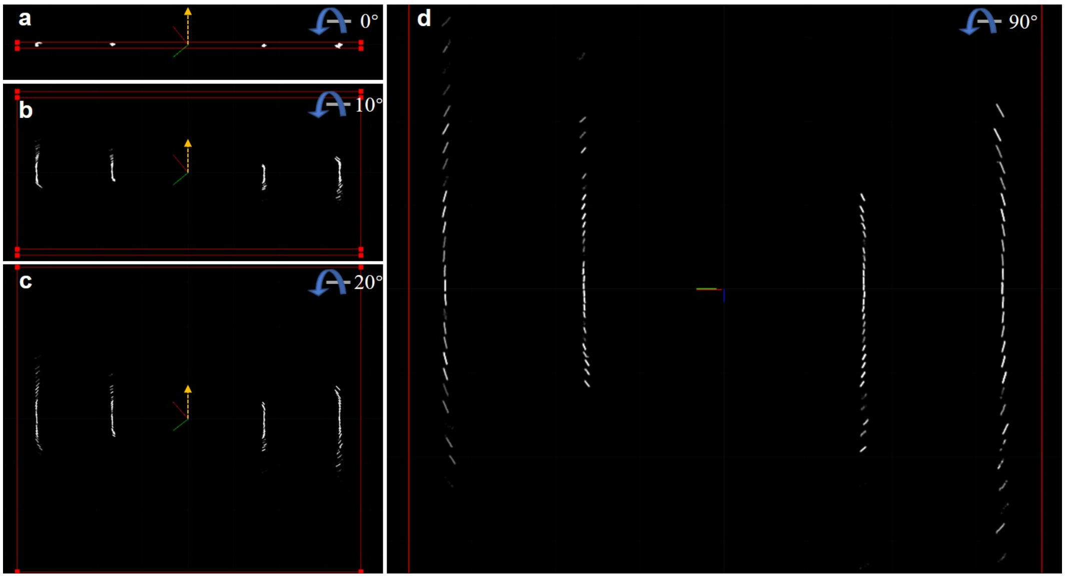

Supplementary Figure 23. Determination of the rotational axis of the plate-like particle in L-CTD. a-d, The modulated arched-shape diffraction spots with rotational intensity distribution. The direction perpendicular to the torsion axis shows the most obvious rotational intensity distribution. The yellow dotted line implies the rotational axis.

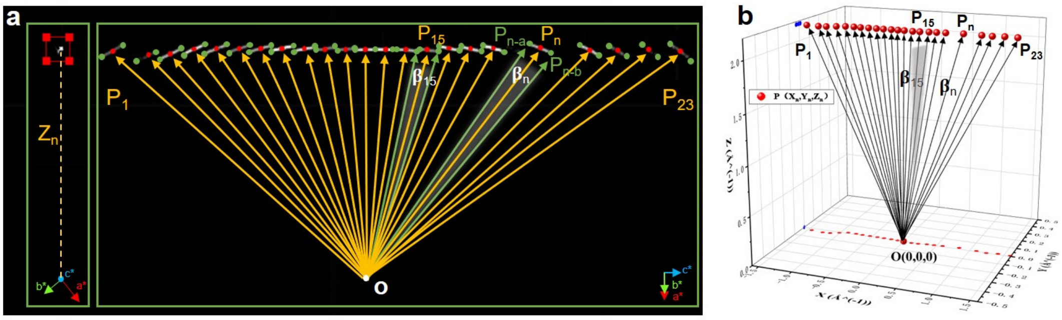

Supplementary Figure 24. Determination of the rotational angle by the intensity distribution of visibly diffraction spots. a, The $\bar{6}\bar{6}l$ diffractions (the enlargement permutation of green rectangle in Fig. 3l in the main text) cut from the reconstructed reciprocal lattice of the plate-like L-CTD are shown in front view (left) and side view (right). P_n (X_n, Y_n, Z_n) represents the Cartesian coordinates of diffraction spots (centers of the diffractions are marked by red dots and the two endpoints are marked by the green dots), and the yellow arrows represent the normal direction of the corresponding lattice planes. **b**, The 3D model diagram of the diffraction spots (P_n). β_n represents the rotational angle between two endpoints of P_n and the origin O. The results of P_n (X_n, Y_n, Z_n) coordinates are listed in Supplementary Table 6, The coordinates of endpoint defined as $P_{n\text{-}a}$ ($X_{n\text{-}a}$, $Y_{n\text{-}a}$, $Z_{n\text{-}a}$) and $P_{n\text{-}b}$ ($X_{n\text{-}b}$, $Y_{n\text{-}b}$, $Z_{n\text{-}b}$) are shown in Supplementary Table 7.

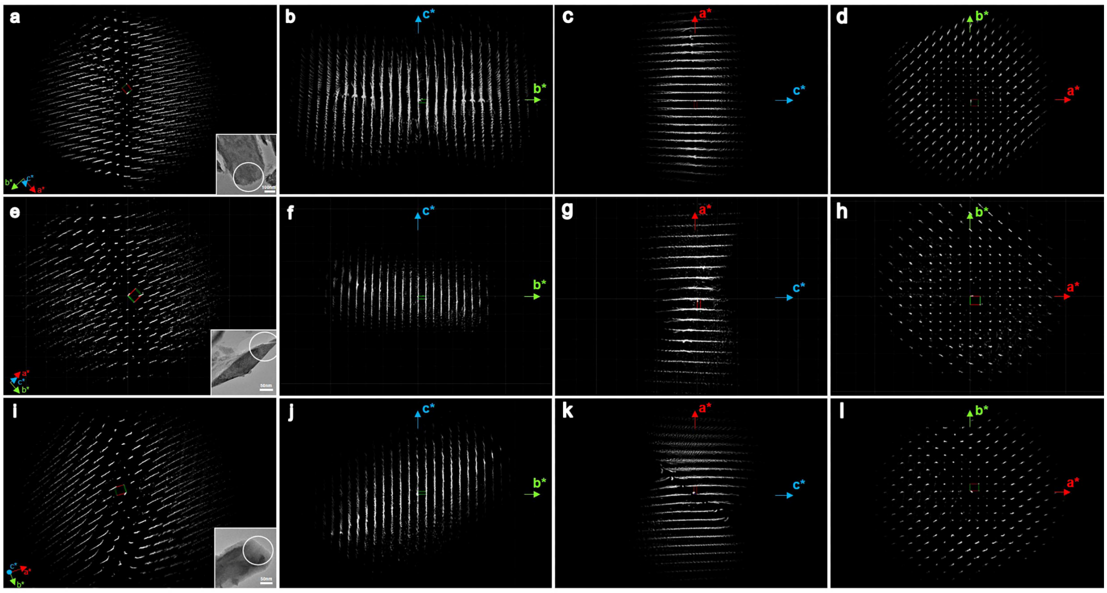

Supplementary Figure 25. 3D ED datasets of other samples in L-CTD. a-l, The whole 3D ED and 3D ED patterns of L-CTD observed from the ***a****-, ***b****- and ***c****-axes, respectively. The tilting ranges are -45.26° to +45.32° for L-CTD$_{-1}$ (**a-d**), -53.95° to +8.03° for L-CTD$_{-2}$ (**e-h**), -52.59° to +34.81° for L-CTD$_{-3}$ (**i-l**), respectively.

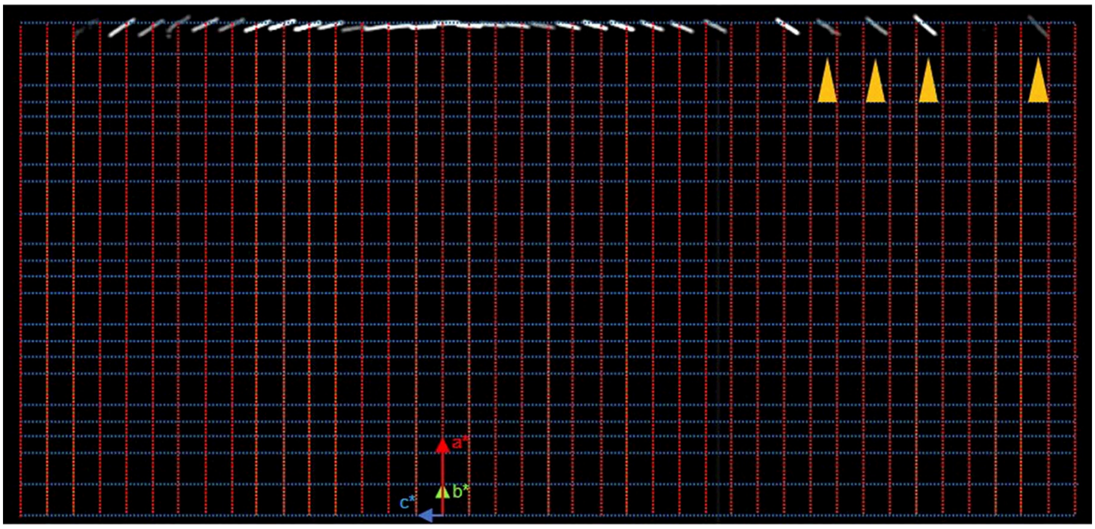

Supplementary Figure 26. The enlargement permutation of the typical arched-shape 53*l* diffraction cut from the reconstructed reciprocal lattice of the plate-like particle in L-CTD. Every electron diffraction spot presents the arched-shape intensity distribution. The yellow arrows indicate the diffraction spots deviated from the reciprocal lattice sites.

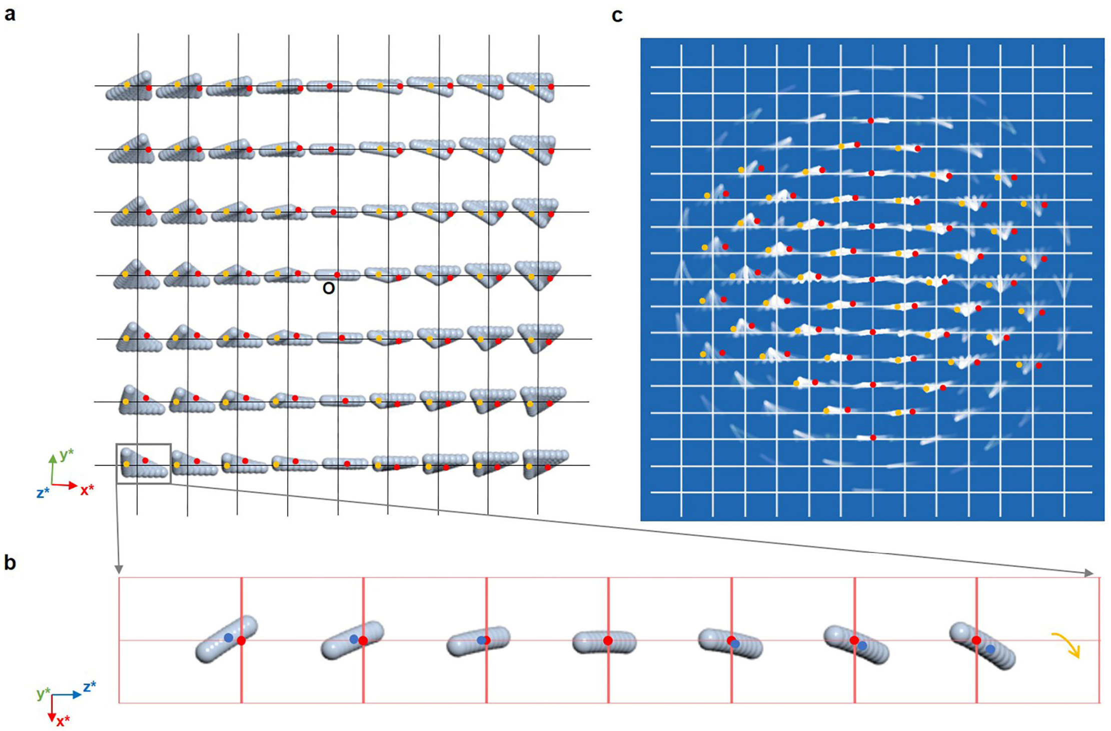

Supplementary Figure 27. Schematic drawing and simulation of the deviation of the diffraction spots from the reciprocal lattice points induced by the crystal lattice torsion. a, The modulated 3D ED model of the twisted crystal. The models were built by rotating the initial lattice of a single crystal by two perpendicular axes corresponding to lattice torsion with small angular steps. The yellow and red dots represent the centers of the first and last arched-shape diffraction in the series of modulated diffraction spots. **b,** The enlargement of a column of high-order arch-shaped diffraction spots (marked by the grey box in **a**). The red dots represent the primary reciprocal lattice sites on the red perpendicular reference lines and the blue dots represent the center of the modulated arched-shape diffraction. The yellow arrow indicates the direction of the diffraction spots shifting caused by the torsional crystal lattice. **c,** The simulation of 3D ED patterns of the twisted CTD crystal. The yellow and red dots represent the center of the first and last diffraction on a series of arched-shape reciprocal-lattice rods. The positional deviations between yellow and red dots can be observed due to the twisting of chiral crystal. In this simulation, eighteen tin dioxide nanocrystals are twisted by 0.6° for each crystal along the direction perpendicular to the $(1\bar{1}0)$ plane.

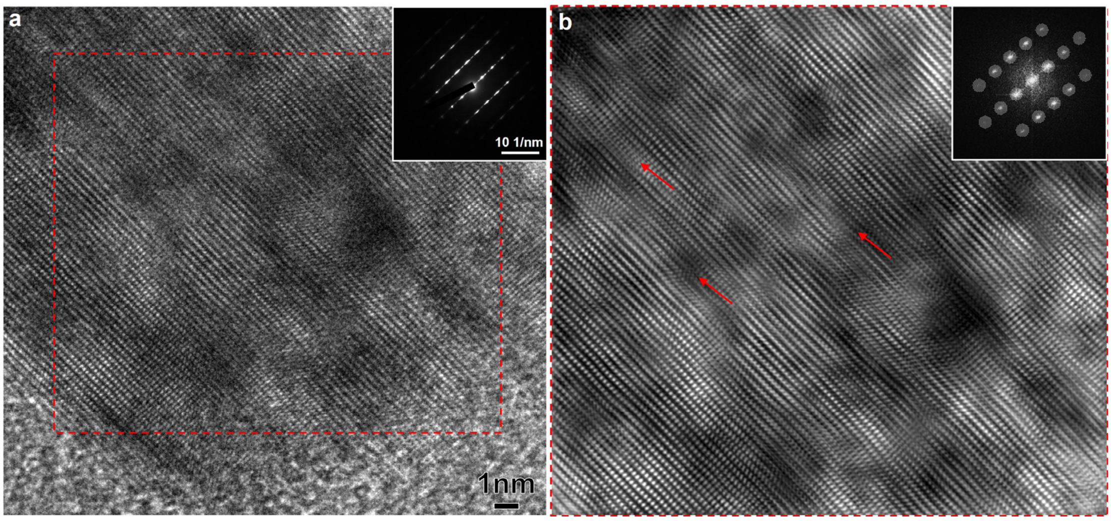

Supplementary Figure 28. High resolution TEM (HRTEM) image and its corresponding Fourier filtered image taken from $[1\bar{1}0]$ axis of the plate-like particle in L-CTD. a, HRTEM image of the plate-like particle in L-CTD and its corresponding SAED pattern of the red dotted rectangle. **b,** The Fourier filtered image obtained by the inverse Fourier transform by the selected diffractions. The red arrows in **b** indicate the dislocations and defects in the L-CTD.

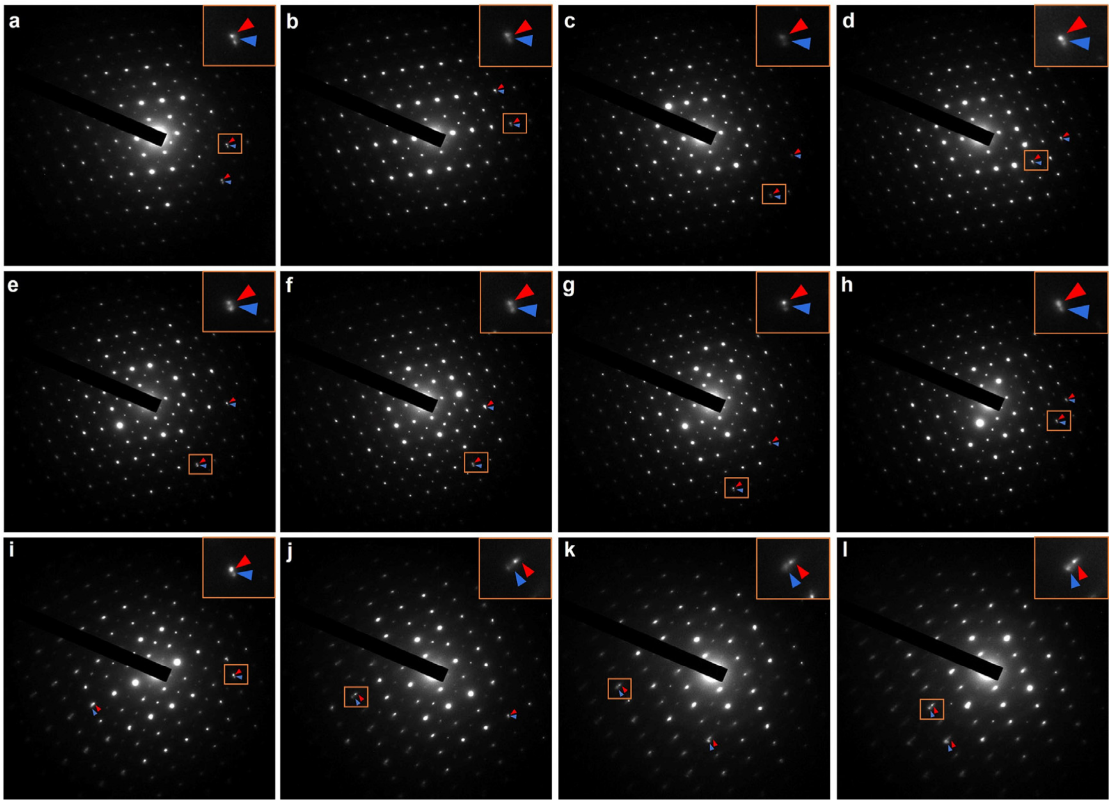

Supplementary Figure 29. The collected ED patterns taken from the series of 3D ED of the plate-like particle in L-CTD. a-l, Series of typical ED patterns obtained during the acquisition of 3D ED data from the plate-like particle. Insets indicate an enlarged view of the characteristic diffraction spots marked by the orange boxes. The red and blue arrows indicate the superposition of two sets of diffraction spots corresponding to the rotational stacked nanosheets in L-CTD samples.

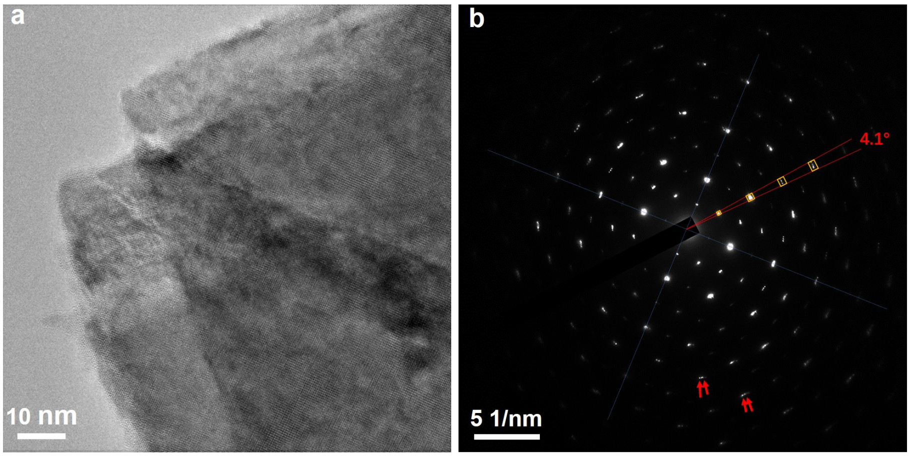

Supplementary Figure 30. HRTEM image and the corresponding SAED pattern taken from [001] zone axis of the plate-like particle in L-CTD. a, The TEM image of overlapped two nanosheets. **b**, The corresponding SAED pattern. The average central angle (4.1°) between the visible diffraction spots to the origin represents the rotational angle between the two nanosheets.

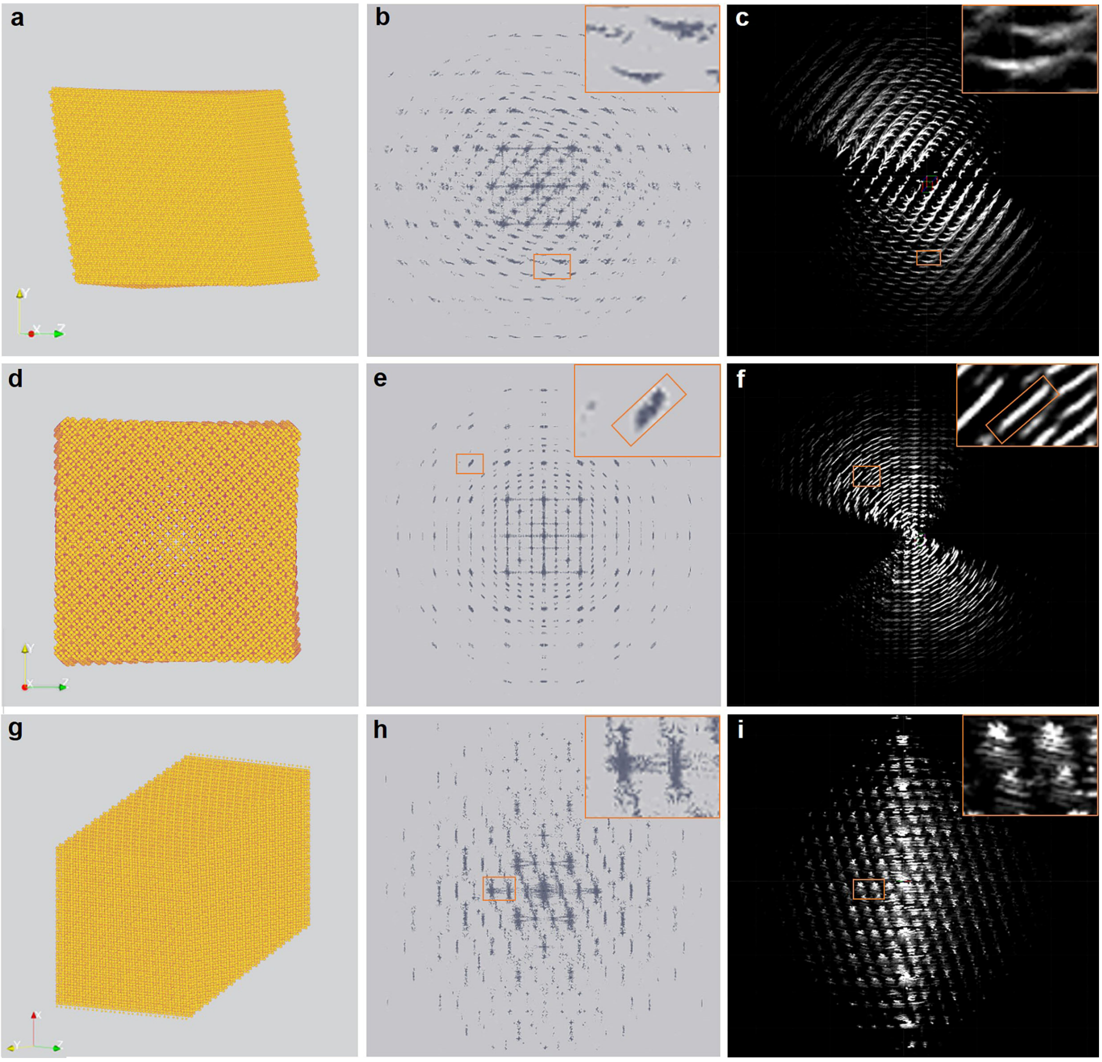

Supplementary Figure 31. a-b, d-e, g-h, Structural model of the twisted crystal of CNM and the corresponding reciprocal space from different orientations. The repeating units of the twisted crystal structural model of CNM are set to be 15, 15, 15 for the *x*-, *y*-, *z*-axes, respectively. The twisted crystal was built by rotating the atoms along the normal of the $(20\bar{4})$ plane. Due to the limited size of the structural model, the twisting angle is enlarged to 0.01425°/ Å to show the modulation in reciprocal space. Insets indicate the typical modulation of arched-shape diffraction spots. **c, f, i,** The corresponding 3D ED patterns of the rod-like particle in D-CNM and the amplification of typical arched-shape reflection from the same direction.

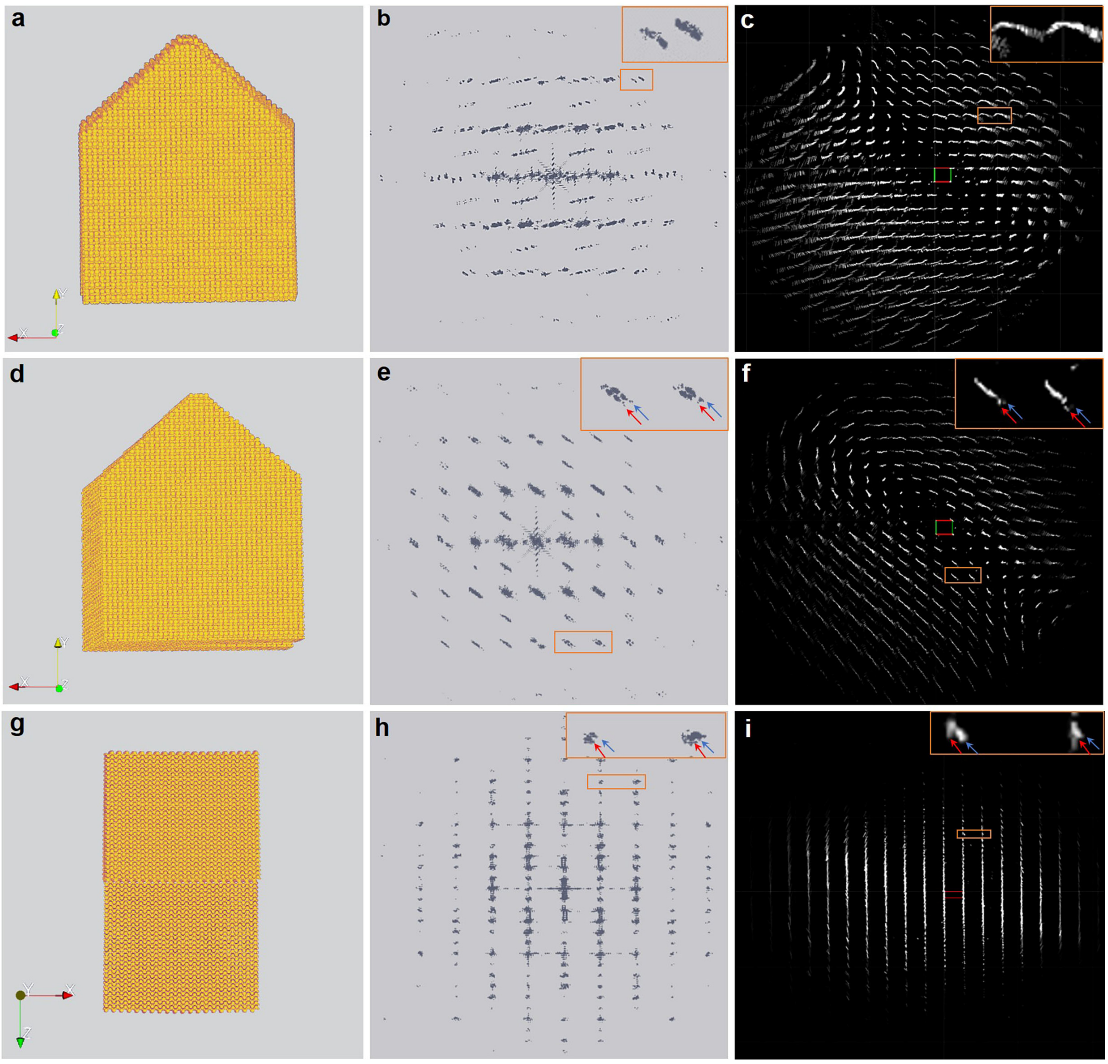

Supplementary Figure 32. a-b, d-e, g-h, Structural model of the chiral hierarchical mesostructure of CTD and the corresponding reciprocal space from different orientations. The assembly was constructed by two layers of twisted nanocrystals with the repeating units of 20, 20, 5 in *x*-, *y*-, *z*-axes, respectively. The CTD was built by rotating the atoms along the normal of the $(1\bar{1}0)$ plane. The twisting angle is set to 0.00955°/Å and the rotation angle of the two layers of crystal is 1.3°. The insets indicate the typical modulation of arched-shape diffraction spots. **c, f, i,** The corresponding 3D ED patterns of the plate-like particle in L-CTD and the amplification of typical arched-shape reflection from the same direction.

2. Supplementary Tables

Supplementary Table 1. Lattice parameters of CNM

	a	*b*	*c*	*β*	Space group
D-CNM (A_1)	9.9 Å	8.8 Å	7.8 Å	114.7°	*C*2/*m* (12)
D-CNM (A_2)	9.6 Å	8.9 Å	7.7 Å	114.2°	*C*2/*m* (12)
D-CNM_{-1}	9.9 Å	8.7 Å	8.0 Å	114.4 °	*C*2/*m* (12)
L-CNM (B_1)	9.6 Å	8.9 Å	7.7 Å	114.2°	*C*2/*m* (12)
L-CNM (B_2)	9.7 Å	8.9 Å	7.7 Å	114.3°	*C*2/*m* (12)
L-CNM_{-1}	9.5 Å	8.6 Å	7.6 Å	114.2°	*C*2/*m* (12)
L-CNM_{-2}	9.9 Å	8.8 Å	7.8 Å	114.8°	*C*2/*m* (12)

Supplementary Table 2. The coordinates of diffraction spots (P_m) in the $h(14-h)0$ reflections cut from the reconstructed reciprocal lattice of the top area (A_1) in the rod-like particle in D-CNM.

	X_m (Å^{-1})	Y_m (Å^{-1})	Z_m (Å^{-1})
P_1	-0.2	0.0	1.1
P_2	0.0	0.0	1.1
P_3	0.2	0.0	1.1
P_4	0.3	0.0	1.1
P_5	0.5	0.0	1.1
P_6	0.6	0.0	1.1
P_7	0.8	0.0	1.1
P_8	1.0	0.0	1.1
P_9	1.1	0.0	1.1
P_{10}	1.3	0.0	1.1
P_{11}	1.5	0.0	1.1
P_{12}	1.6	0.0	1.1

Supplementary Table 3. The endpoint coordinates ($P_{m\text{-}a}$ and $P_{m\text{-}b}$) of the intensity distribution of $h(14-h)0$ reflections (P_m) cut from the reconstructed reciprocal lattice of the top area (A_1) in the rod-like particle in D-CNM.

	$X_{m\text{-}a}$ (Å^{-1})	$Y_{m\text{-}a}$ (Å^{-1})	$Z_{m\text{-}a}$ (Å^{-1})
$P_{1\text{-}a}$	-0.3	0.0	1.1
$P_{2\text{-}a}$	-0.1	0.0	1.1
$P_{3\text{-}a}$	0.1	0.0	1.1
$P_{4\text{-}a}$	0.2	0.0	1.1
$P_{5\text{-}a}$	0.4	0.0	1.1
$P_{6\text{-}a}$	0.6	0.0	1.1
$P_{7\text{-}a}$	0.7	0.0	1.1
$P_{9\text{-}a}$	1.0	0.0	1.1

	$X_{m\text{-}b}$ (Å^{-1})	$Y_{m\text{-}b}$ (Å^{-1})	$Z_{m\text{-}b}$ (Å^{-1})
$P_{1\text{-}b}$	-0.1	0.0	1.1
$P_{2\text{-}b}$	0.1	0.0	1.1
$P_{3\text{-}b}$	0.2	0.0	1.1
$P_{4\text{-}b}$	0.4	0.0	1.1
$P_{5\text{-}b}$	0.6	-0.1	1.1
$P_{6\text{-}b}$	0.7	0.0	1.1
$P_{7\text{-}b}$	0.9	0.0	1.1
$P_{9\text{-}b}$	1.2	-0.1	1.1

Supplementary Table 4. The average value of the rotational angles (α_m) calculated by the end points of intensity distribution from the $h(14-h)0$ reflections. Source data are provided as a Source Data file.

	rotational angle (α_m)
α_1	8.5°
α_2	11.6°
α_3	7.5°
α_4	8.4°
α_5	7.8°
α_6	6.2°
α_7	4.6°
α_9	5.1°
$\overline{\alpha_m}$	7.5°

$$\alpha_m = cos^{-1} \frac{\overrightarrow{OP_{m-a}} * \overrightarrow{OP_{m-b}}}{|\overrightarrow{OP_{m-a}}| * |\overrightarrow{OP_{m-b}}|} \quad (1)$$

Supplementary Table 5. Lattice parameters of L-CTD

	a	*b*	*c*	Space group
L-CTD	4.8 Å	5.9 Å	16.1 Å	*Pbcn*
L-CTD_{-1}	4.8 Å	5.8 Å	16.0 Å	*Pbcn*
L-CTD_{-2}	4.8 Å	5.7 Å	16.0 Å	*Pbcn*
L-CTD_{-3}	4.7 Å	5.7 Å	16.0 Å	*Pbcn*

Supplementary Table 6. The coordinates of diffraction spots (P_n) in the $\overline{6}\overline{6}l$ reflections cut from the reconstructed reciprocal lattice of the plate-like particle in L-CTD.

	X_n (Å^{-1})	Y_n (Å^{-1})	Z_n (Å^{-1})
P_1	-1.6	0.0	2.4
P_2	-1.4	0.0	2.4
P_3	-1.3	0.0	2.4
P_4	-1.1	0.0	2.4
P_5	-1.0	0.0	2.4
P_6	-0.9	0.0	2.4
P_7	-0.7	0.0	2.4
P_8	-0.6	0.0	2.4
P_9	-0.5	0.0	2.4
P_{10}	-0.3	0.0	2.4
P_{11}	-0.2	0.0	2.4
P_{12}	-0.1	0.0	2.4
P_{13}	0.0	0.0	2.4
P_{14}	0.1	0.0	2.4
P_{15}	0.3	0.0	2.4
P_{16}	0.4	0.0	2.4
P_{17}	0.5	0.0	2.4
P_{18}	0.7	0.0	2.4
P_{19}	1.4	0.0	2.4
P_{20}	1.2	0.0	2.4
P_{21}	1.4	0.0	2.4
P_{22}	1.6	0.0	2.4
P_{23}	1.8	0.0	2.4

Supplementary Table 7. The endpoint coordinates ($P_{n\text{-}a}$ and $P_{n\text{-}b}$) of the intensity distribution of $\overline{6}\overline{6}l$ reflections (P_n) cut from the reconstructed reciprocal lattice in the plate-like L-CTD.

	$X_{n\text{-}a}$ (Å^{-1})	$Y_{n\text{-}a}$ (Å^{-1})	$Z_{n\text{-}a}$ (Å^{-1})
$P_{1\text{-}a}$	-1.7	0.0	2.4
$P_{2\text{-}a}$	-1.5	-0.1	2.4
$P_{3\text{-}a}$	-1.3	-0.1	2.4
$P_{4\text{-}a}$	-1.2	-0.1	2.4
$P_{5\text{-}a}$	-1.1	0.0	2.4
$P_{6\text{-}a}$	-0.9	0.0	2.4
$P_{7\text{-}a}$	-0.8	0.0	2.4
$P_{8\text{-}a}$	-0.7	0.0	2.4
$P_{9\text{-}a}$	-0.5	0.0	2.4
$P_{10\text{-}a}$	-0.4	0.0	2.4
$P_{11\text{-}a}$	-0.3	0.0	2.4
$P_{12\text{-}a}$	-0.2	0.0	2.4
$P_{13\text{-}a}$	0.0	0.0	2.4
$P_{14\text{-}a}$	0.1	0.0	2.4
$P_{15\text{-}a}$	0.2	0.0	2.4
$P_{16\text{-}a}$	0.3	0.0	2.4
$P_{17\text{-}a}$	0.5	0.0	2.4
$P_{18\text{-}a}$	0.6	0.0	2.4
$P_{19\text{-}a}$	0.9	0.0	2.4
$P_{20\text{-}a}$	1.2	0.0	2.4
$P_{21\text{-}a}$	1.3	0.0	2.4
$P_{22\text{-}a}$	1.5	0.0	2.4
$P_{23\text{-}a}$	1.7	0.0	2.4

	$X_{n\text{-}b}$ (Å^{-1})	$Y_{n\text{-}b}$ (Å^{-1})	$Z_{n\text{-}b}$ (Å^{-1})
$P_{1\text{-}b}$	-1.5	0.0	2.4
$P_{2\text{-}b}$	-1.3	0.0	2.4
$P_{3\text{-}b}$	-1.2	0.0	2.4
$P_{4\text{-}b}$	-1.1	0.0	2.4
$P_{5\text{-}b}$	-0.9	0.0	2.4
$P_{6\text{-}b}$	-0.8	0.0	2.4
$P_{7\text{-}b}$	-0.7	0.0	2.4
$P_{8\text{-}b}$	-0.5	0.0	2.4
$P_{9\text{-}b}$	-0.4	0.0	2.4
$P_{10\text{-}b}$	-0.3	0.0	2.4
$P_{11\text{-}b}$	-0.2	0.0	2.4
$P_{12\text{-}b}$	0.0	0.0	2.4
$P_{13\text{-}b}$	0.1	0.0	2.4
$P_{14\text{-}b}$	0.2	0.0	2.4
$P_{15\text{-}b}$	0.3	0.0	2.4
$P_{16\text{-}b}$	0.4	0.0	2.4
$P_{17\text{-}b}$	0.6	0.0	2.4
$P_{18\text{-}b}$	0.8	0.0	2.4
$P_{19\text{-}b}$	1.0	0.0	2.4
$P_{20\text{-}b}$	1.3	0.0	2.4
$P_{21\text{-}b}$	1.4	0.0	2.4
$P_{22\text{-}b}$	1.6	0.0	2.4
$P_{23\text{-}b}$	1.8	0.0	2.4

Supplementary Table 8. The average value of the rotational angles (β_n) calculated by the endpoints coordinates of intensity distribution from the $\bar{6}\bar{6}l$ reflections. Calculation of the average value of the rotational angles was achieved by visibly diffractions in the $\bar{6}\bar{6}l$ reflections, where the origin *O* coordinate can be defined as (0,0,0). $P_{n\text{-}a}$ ($X_{n\text{-}a}$, $Y_{n\text{-}a}$, $Z_{n\text{-}a}$) and $P_{n\text{-}b}$ ($X_{n\text{-}b}$, $Y_{n\text{-}b}$, $Z_{n\text{-}b}$) are the two endpoints coordinates. Source data are provided as a Source Data file.

	rotational angles (β_n)
β_1	2.7°
β_2	2.5°
β_3	2.7°
β_4	3.0°
β_5	2.9°
β_6	2.8°
β_7	2.9°
β_8	2.9°
β_9	2.9°
β_{10}	2.9°
β_{11}	2.8°
β_{12}	5.2°
β_{13}	2.8°
β_{14}	3.0°
β_{15}	2.8°
β_{16}	2.8°
β_{17}	2.8°
β_{18}	3.8°
β_{19}	2.7°
β_{20}	2.5°
β_{21}	2.3°
β_{22}	2.1°
β_{23}	2.2°

$$\beta_n = \cos^{-1} \frac{\overrightarrow{OP_{n-a}} * \overrightarrow{OP_{n-b}}}{|\overrightarrow{OP_{n-a}}| * |\overrightarrow{OP_{n-b}}|} \tag{2}$$
