# Supplementary material for: Synchronous quantitative analysis of chiral mesostructured inorganic crystals by 3D electron diffraction tomography
Source: Nat Commun. 2022 Sep 29;13:5718. doi: 10.1038/s41467-022-33443-1 (PMC9522932; doi:10.1038/s41467-022-33443-1)
Supplement: Supplementary file 3 — Description of Additional Supplementary Files [file 41467_2022_33443_MOESM3_ESM.pdf]

Supplementary Movie 1: Reciprocal space of Au single crystal structure model was calculated by the Fourier transform corresponding to Supplementary Figure 1.

Supplementary Movie 2: Reciprocal space of bent Au crystal structure model was calculated by the Fourier transform corresponding to Supplementary Figure 2.

Supplementary Movie 3: Reciprocal space of twisted Au crystal structure model was calculated by the Fourier transform corresponding to Supplementary Figure 4.

Supplementary Movie 4: Reciprocal space of helically stacked Au nanocrystals crystal structure model was calculated by the Fourier transform corresponding to Supplementary Figure 6.

Supplementary Movie 5: Reciprocal space of chiral hierarchical mesostructure formed by the stacked of primary twisted nanocrystals structure model was calculated by the Fourier transform corresponding to Supplementary Figure 7.

Supplementary Movie 6: Experimental 3D ED data of the rod-like particle in D-CNM (Fig. 2g). The movie was obtained via the “3D VIEW” window of EDT-PROCESS software by rotating the reconstructed 3D reciprocal lattice every 1° or 5° along the *x*, *y*, *z*-axes, respectively.

Supplementary Movie 7: The whole dataset of 3D ED of the plate-like particle in L-CTD (Fig. 3f). The movie was obtained via the “3D VIEW” window of EDT-PROCESS software by rotating the reconstructed 3D reciprocal lattice every 1° along the *x*, *y*, *z*-axes, respectively.
